# α-Mangostin Inhibits the Activation of Myofibroblasts via Downregulation of Linc-ROR-Mediated TGFB1/Smad Signaling

**DOI:** 10.3390/nu15061321

**Published:** 2023-03-08

**Authors:** Yu-Hsien Lee, Pei-Ling Hsieh, Shih-Chi Chao, Yi-Wen Liao, Chia-Ming Liu, Cheng-Chia Yu

**Affiliations:** 1 School of Dentistry, Chung Shan Medical University, Taichung 40201, Taiwan; 2 Department of Dentistry, Chung Shan Medical University Hospital, Taichung 40201, Taiwan; 3 Department of Anatomy, School of Medicine, China Medical University, Taichung 404333, Taiwan; 4 Institute of Oral Sciences, Chung Shan Medical University, Taichung 40201, Taiwan; 5 Department of Medical Research and Education, Lo-Hsu Medical Foundation, Lotung Poh-Ai Hospital, Yilan 265, Taiwan; 6 Department of Medical Research, Chung Shan Medical University Hospital, Taichung 40201, Taiwan

**Keywords:** oral submucous fibrosis, myofibroblast, α-mangostin, LincROR

## Abstract

Oral submucous fibrosis (OSF) is a premalignant disorder and persistent activation of myofibroblasts is implicated in this pathological progression. Increasing attention has been addressed towards non-coding RNA-regulated myofibroblasts activities and the effects of phytochemicals on non-coding RNA modulation are of great importance. In the present study, we examined the anti-fibrosis property of α-mangostin, a xanthone isolated from the pericarp of mangosteen. We found that α-mangostin exhibited inhibitory potency in myofibroblast activities and expression of fibrosis markers at the concentrations that caused neglectable damage to normal cells. Apart from the downregulation of TGF-β1/Smad2 signaling, we found that α-mangostin attenuated the expression of long non-coding RNA LincROR as well. Our results demonstrated that the effects of α-mangostin on myofibroblast activation were reverted when LincROR was overexpressed. Additionally, we showed the expression of LincROR in OSF specimens was elevated and silencing of LincROR successfully attenuated myofibroblast characteristics and TGF-β1/Smad2 activation. Taken together, these findings indicated that the anti-fibrosis effects of α-mangostin merit consideration and may be due to the attenuation of LincROR.

## 1. Introduction

Oral submucous fibrosis (OSF) is a potentially malignant disorder that was first reported by Schwartz in 1952. This chronic scarring disease is characterized by juxta-epithelial inflammation and collagen deposition, leading to difficulty in mouth opening. Aside from the development of vertical fibrous bands, patients often have burning sensations, ulceration, and pain. Moreover, its malignant transformation rate is around 5% [1] and the hazard rate ratio of tongue lesions is higher than buccal lesions [2]. The etiology of OSF is multifactorial, such as genetic susceptibility [3], human papillomavirus (HPV) infection [4], consumption of tobacco, alcohol, and areca nut [5]. Among these factors, the habit of areca nut chewing has been postulated as the main causative event. It has been demonstrated that collagen phagocytosis of buccal mucosal fibroblasts (BMFs) was reduced in response to areca nut alkaloids stimulation [6]. Moreover, the expression of tissue inhibitor of metalloproteinase-1 (TIMP-1) was elevated and matrix metalloproteinase 2 (MMP-2) was inhibited in BMFs treated with arecoline, a major alkaloid ester in areca nut [7,8]. These results suggested areca nut chewing resulted in an impairment of matrix degradation in BMFs. Furthermore, constituents of areca nut were found to activate the transforming growth factor-β1 (TGF-β1)/Smad2 pathway in epithelial cells [9] and increase the transdifferentiation of gingival fibroblasts into myofibroblasts [10]. 

As the pivotal cells to secrete collagen, myofibroblasts have long been a subject of investigation for OSF study in terms of their origin, dynamics, and biological mechanisms. It is well-accepted that fibroblasts differentiate into highly contractile myofibroblasts following injury in order to remodel the extracellular matrix (ECM) scaffold and maintain the structural integrity [11]. In addition to resident fibroblasts, cells that undergo epithelial-to-mesenchymal transition (EMT) appear to be a potential source of myofibroblasts in liver or kidney fibrosis diseases [12,13]. EMT confers cells with mesenchymal phenotype, including migratory and invasive properties. Various transcription factors, such as Slug, Snail, and Twist, are known as repressors of E-cadherin and inducers of EMT [14]. It has been shown that arecoline stimulation elicited the expression of Slug, Snail, and Twist in BMFs [15,16]. Moreover, Slug and Snail were found to mediate myofibroblast transdifferentiation through directly binding to the E-box of type I collagen or alpha-smooth muscle actin (α-SMA) promoter, respectively [15,16]. Snail also has been demonstrated to bind to the interleukin-6 (IL-6) promoter, leading to an increase in α-SMA and type I collagen in fBMFs [17]. TGF-β1 is essential for induction of EMT and fibrosis [8], and several natural compounds have been exploited to suppress OSF via inhibition of TGF-β1 signaling [18,19]. A better understanding pertaining to the inhibitory effect of natural compounds on myofibroblast activation through regulation of the TGF-β1 pathway is of great importance. 

Over the past decades, numerous studies have revealed that non-coding RNAs serve significant roles in the modulation of myofibroblasts. These non-protein-coding RNAs can be categorized into short (<200 bp; e.g., microRNAs) and long non-coding RNAs (lncRNAs; >200 bp) based on their length. MicroRNAs interact and restrict their target genes via direct binding to sequences located in the 3’ untranslated regions (3’UTR) of mRNAs [20]. LncRNAs can be divided into three categories, including antisense that are transcribed across protein-coding genes on the reverse strand, pseudogenes that are non-translated protein-coding genes and long intergenic non-coding RNAs (lincRNAs) that are located between protein-coding genes [21]. The functions and mechanisms of lncRNAs are more complex. For instance, lncRNAs can act as decoys/competing endogenous RNAs, and bind to their target genes or even microRNAs to downregulate their action [22]. Up to now, a great effort has been made to address the implication of microRNAs in the pathogenesis of OSF (see review [23]). Accumulating research also revealed the significance of lncRNAs in the pathogenesis of OSF. For example, upregulation of lncRNA H19 in BMFs was reported as a result of areca-nut-induced TGF-β/Samd activation, which impeded the suppressive property of microRNA-29b on type I collagen [24]. Another lncRNA, LINC00974, also has been shown to promote arecoline-elicited myofibroblast transdifferentiation via the TGF-β pathway [25] and inhibition of LINC00974 by natural compound successfully mitigated myofibroblast activities [26]. Unlike genic lncRNAs share sequences with coding loci, lincRNAs constitute more than half of lncRNA transcripts but they do not overlap annotated coding genes [27]. To date, the regulation and function of lincRNAs remain an area of interest.

Numerous bioactive substances and phytochemicals have been demonstrated to exert pharmacological effects and improve various kinds of diseases, such as cancer or fibrosis disorders. α-, β- and γ-mangostin are xanthones isolated from mangosteen (*Garcinia mangostana*) and they exhibit a wide range of biological properties. α-mangostin has been revealed to induce apoptosis and cell cycle arrest of oral cancer cells [28]. A study using mucoadhesive film containing α-mangostin also demonstrated the anti-cancer effect on oral cancer cells and anti-inflammatory activities in RAW 264.7 cells [29]. In addition to anti-tumor effects, α-mangostin also possesses inhibitory properties. For instance, it has been revealed to attenuate the bleomycin-induced pulmonary fibrosis [30] and improve cardiac hypertrophy and fibrosis in diabetic rats [31]. Moreover, α-mangostin has been demonstrated to reduce the acetaldehyde-induced liver fibrosis by inhibiting myofibroblast transdifferentiation of hepatic stellate cells along with decreased expression of TGF-β and increased anti-oxidant capacity [32]. Similarly, it markedly suppressed the TGF-β-induced myofibroblast differentiation and oxidative stress in cardiac fibroblasts [33]. Nevertheless, whether α-mangostin can ameliorate oral fibrogenesis has not been elucidated.

In this study, we first examined the suppressive effects of α-mangostin on cell viability, myofibroblast activities, TGF-β1/Smad2 pathway and type I collagen. Furthermore, we showed the expression of lincRNA-RoR (LincROR) was downregulated in α-mangostin-treated cells. Linc-RoR is implicated in tumorigenesis and was found to be overexpressed in oral cancer with a strong association with poor prognosis [34]. However, whether the level of lincRNA-RoR was also upregulated in precancerous OSF has not been investigated. Herein, we assessed the expression of lincRNA-RoR in OSF specimens and demonstrated its critical function in the α-mangostin-mediated myofibroblast inhibition.

## 2. Materials and Methods

### 2.1. Tissue Collection, Primary Culture and Reagents

All procedures were followed the protocol that is granted from IRB of Chung Shan Medical University Hospital (approval number: CSMUH No. CS18124). After acquiring patients’ consent, the healthy and OSF tissues obtained from surgery were immediately immersed in phosphate buffered saline (PBS) for primary culture or liquid nitrogen for the subsequent quantification of LincROR expression. Normal buccal mucosal fibroblasts (BMFs) and fibrotic buccal mucosal fibroblasts (fBMFs) were isolated from fresh healthy buccal mucosa and OSF tissues, respectively. In brief, the tissues were cut into small pieces (0.5–1.0 mm^2^) and incubated with trypsin-EDTA (0.05%) at 37 °C for 30 min. After centrifugation at 1200 rpm, the tissue pellets were plated into a 10-cm culture dish with a growth medium at 37 °C/5% CO_2_. After 7–14 days of incubation, cells with a spindle shape that crawled out from the tissues were harvested and routinely maintained in growth medium which was composed of the following components: 90% Dulbecco’s Modified Eagle Medium (DMEM), 10% fetal bovine serum (FBS), 100 U/mL penicillin and 100 μg/mL streptomycin. Cells between the third and eighth passages were used in this study. All reagents were purchased from Sigma (St. Louis, MO, USA) unless stated otherwise.

### 2.2. Cell Proliferation and Survival Assay

Cells were seeded into a 96-well plate at a density of 1.0 × 10^4^ cells/well for 24 h of incubation, and then replaced with a fresh culture medium containing a series concentration of α-mangostin (0~80 μM) for another 24 h of incubation. Subsequently, the proliferation rate and IC_50_ value were estimated using 3-(4,5-Dimethyl-2- thiazolyl)-2,5-diphenyltetrazolium bromide (MTT) assay, according to the manufacturer’s protocol (Sigma-Aldrich, St. Louis, MO, USA). The absorbance at 570 nm was determined using a microplate reader (Molecular Devices, San Jose, CA, USA).

### 2.3. Collagen Gel Contraction Assay

We embedded cells to type I collagen gel solution (2 ng/mL) at a density of 2.0 × 10^5^ cells/well in a 24-well plate. We mixed the cells and gel solution gently and incubated the plate at 37 °C for 2 h to allow the polymerization of gels. Then, we added 0.5 mL culture medium in each well to cover the gel and incubated for another 48 h for gel contraction by cells. The contraction index was quantified using ImageJ software (NIH, Bethesda, MD, USA) [35].

### 2.4. Transwell Migration Assays

1 × 10^5^ cells suspended in 150 μL serum-free medium were added into the Transwell inserts (Corning, Acton, MA, USA), and then we added the 750 μL completed growth medium (with 10% FBS) into the lower chamber to create a chemo-gradient that attracted cell migration. After incubation for 24 h, cells were fixed with cold-100% methanol and stained with 0.1% crystal violet. Then, the non-migrated cells on the topside of the Transwell insert were gently removed using a cotton swab. We counted the number of migrated cells on the underside from five randomly selected fields under the microscope.

### 2.5. Enzyme-Linked Immunosorbent Assay (ELISA)

According to the manufacturer’s instructions, the amount of TGF-β in the culture medium secreted from fBMFs treated with 0-4 μM α-mangostin was to be measured by Human TGF-β1 ELISA Kit (Abcam, Cambridge, UK). The absorbance at 450 nm was determined using a SpectraMax M5 microplate reader (Molecular Devices, San Jose, CA, USA).

### 2.6. RNA-Sequencing

Total RNA from three independent fBMFs treated with or without α-mangostin was extracted using TRIzol™ Reagent according to the manufacturer’s protocol (Invitrogen Life Technologies, Carlsbad, CA, USA). The RNA quality of each cell was ensured by the manufacturer of Genomics Inc. After RNA-seq library preparation and construction, the changes in transcriptome of cells were analyzed using the FPKM method (fragments per kb of transcript per million mapped reads) on Illumina HiSeq platform (HiSeq2500 platform, Illumina, San Diego, CA, USA) as previously described [17].

### 2.7. Real-Time Quantitative Polymerase Chain Reaction (Qrt-PCR)

Total RNA was extracted from tissues and cells using TRIzol™ Reagent according to the manufacturer’s protocol (Invitrogen Life Technologies, Carlsbad, CA, USA). Complementary DNA (cDNA) synthesis and quantitative polymerase chain reactions were conducted using Superscript III first-strand synthesis system (Invitrogen Life Technologies, Carlsbad, CA, USA) and ABI StepOne™ Real-Time PCR Systems (ThermoFisher Scientific, Carlsbad, CA, USA), respectively. Primer sequences used were listed as follows: LincROR, 5′-CTGGCTTTCTGGTTTGACG-3′ (forward), 5′-CAGGAGGTTACTGGACTTGGAG-3′ (reverse); GAPDH, 5′-CTCATGACCACAGTCCATGC-3′ (forward) and 5′-TTCAGCTCTGGGATGACCTT-3′ (reverse). The expression of LincROR related to GAPDH was calculated using the delta Ct and comparative methods.

### 2.8. Western Blot Analysis

The whole cell lysates were obtained using 1×RIPA buffer with protease and phosphatase inhibitor cocktail (Abcam, Cambridge, MA, UK). The total protein concentration of each sample was quantified according to the Bradford assay (Bio-Rad Laboratories Inc., Hercules, CA, USA). Cell lysates containing 20 μg protein were loaded onto 10% SDS-polyacrylamide gel and transferred onto the PVDF membrane (Millipore, Billerica, MA, USA). The PVDF membranes were blocked in 5% bovine serum albumin (BSA) at room temperature for 1 h, followed by incubation with primary antibodies at 4 °C for 16 h and HRP-conjugated secondary antibodies at room temperature for 1 h. The chemical luminescence of each immunoreactive band was developed by adding the ECL chemiluminescent agent and captured by using a LAS-1000plus Luminescent Image Analyzer (GE Healthcare Biosciences, Piscataway, NJ, USA). The primary antibodies are listed as follows: anti-α-SMA (Abcam), anti-COL1A1 (Abcam), anti-p-Smad2 (Cell Signaling Technology, Danvers, MA, USA), anti-Smad (Cell Signaling), and anti-GAPDH (GeneTex Inc., Irvine, CA, USA).

### 2.9. Lentiviral-Mediated Silencing and Overexpression of Lncrna-ROR

To construct the lentivirus-LincROR silencing vector (pLV-Sh-LincROR), oligonucleotide sequence shRNA that targets human lincROR was synthesized and ligated into pLV-RNAi vector following the manufacturer’s protocol (Biosettia, San Diego, CA, USA). The target sequences for LincROR are listed as follows: Sh-LincROR-1 5′-AAAAGGAAACTGGCAATGTTGAATTGGATCCAATTCAACATTGCCAGTTTCC-3′; Sh-LincROR-2 5′-AAAAGGAGGATGCAGAGAAATTATTGGATCCAATAATTTCTCTGCATCCTCC-3′. To construct the lentivirus-LincROR overexpressing vector (pLV-LincROR-cDNA), the full-length LincROR cDNA was amplified using RT-PCR and then cloned into a pLV-EF1a-MCS-IRES-Puro vector (BioSettia). The pLV-Sh-LincROR or pLV-LincROR-cDNA vector was co-transfected with the packaging and envelope vectors into 293T cells using Lipofectamine 2000, according to the manufacturer’s protocol (LF2000, Invitrogen, Calsbad, CA, USA) to produce lentiviral particles. Overexpression and knockdown of LincROR were conducted by infecting cells with lentiviral particles carrying full-length LincROR or shRNA sequences targeting LncRNA-ROR, respectively [24].

### 2.10. Statistical Analysis

Data were obtained from at least three individual experiments and were presented as mean ± standard deviation. A Student’s *t*-test or analysis of variance (ANOVA) were performed to determine the statistical significance of the difference using Statistical Package of Social Sciences software (version 13.0, SPSS, Inc., Chicago, IL, USA).

## 3. Results

### 3.1. α-Mangostin Reduces the Cell Viability of Fbmfs and Has Minimal Effect on Normal Oral Cells

**Figure 1:**
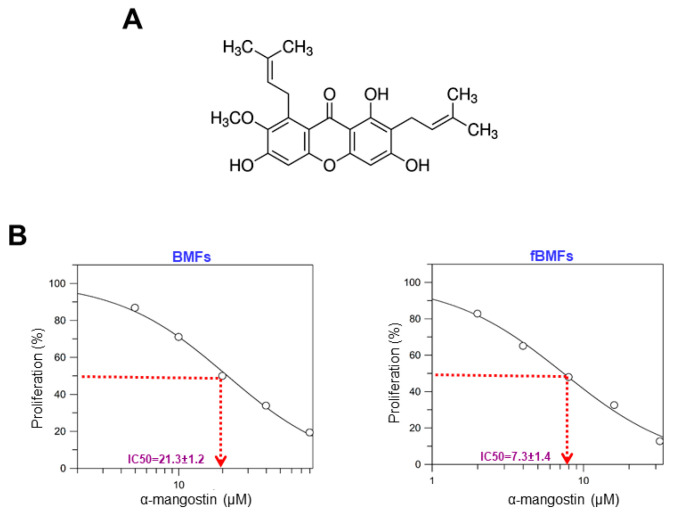
α-mangostin inhibited the proliferation rate of fBMFs without causing damage to normal BMFs. (**A**) The chemical structure of α-mangostin. (**B**) The cytotoxicity effect of α-mangostin in normal buccal mucosal fibroblasts (BMFs) and fibrotic BMFs (fBMFs). Cell survival/viability of BMFs (**left** panel) and fBMFs (**right** panel) were determined by MTT assay. IC_50_ values were calculated by GraFit software.

To determine the cytotoxic effect of α-mangostin (Figure 1A) on normal BMFs and fibrotic BMFs (fBMFs)-derived from OSF tissues, cell proliferation rate was measured after treatment of α-mangostin with various concentrations for 24 h using an MTT assay. In both BMFs and fBMFs, a concentration-dependent inhibitory effect on cell survival was observed. The IC_50_ values for α-mangostin in BMFs and fBMFs were 21.3 ± 1.2 and 7.3 ± 1.4 μM, respectively (Figure 1B). These results showed that a lower concentration (0–4 μM) of α-mangostin was sufficient to reduce the cell viability of fBMFs without causing severe damage to normal oral cells.

### 3.2. α-Mangostin Suppresses the Myofibroblast Activation of fBMFs

**Figure 2:**
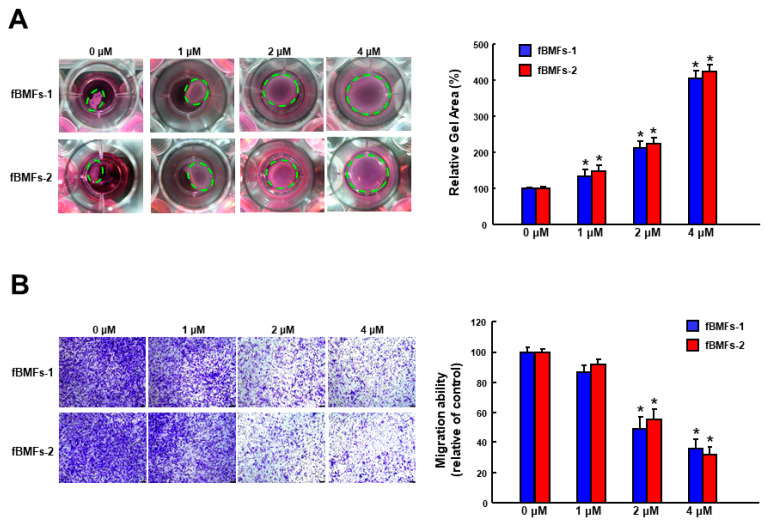
α-mangostin inhibits the myofibroblast activities of fBMFs. (**A**) The contractile capacity of fBMFs was determined by collagen gel contraction assay (three replicates for each concentration). Images of gels were captured, and gel areas (dotted circles) were calculated by ImageJ software. (**B**) fBMFs were treated with the indicated concentration of α-mangostin followed by transwell migration assay. The experiments were repeated for three times and data from a representative experiment were presented. Results are means ± SD. * *p* < 0.05 compared to control.

Aside from cell proliferation, activated myofibroblasts will migrate to the wound area to restore tissue integrity and close the wound. Hence, we examined the effects of α-mangostin on collagen gel contraction ability, which is a well-established assay to investigate fibroblast-matrix interactions by Bell et al. [36]. As shown in Figure 2A, the relative gel area was increased in fBMFs treated with α-mangostin in a concentration-dependent manner, suggesting a higher dose of α-mangostin relieved the contractile activity of fBMFs. Additionally, fBMFs were subjected to transwell migration assay, and the result showed that α-mangostin dose-dependently downregulated the migration capacity of fBMFs (Figure 2B). 

### 3.3. Incubation of α-Mangostin Downregulates the Expression of TGF-β1 Signaling, Myofibroblast Marker, and LincROR

**Figure 3:**
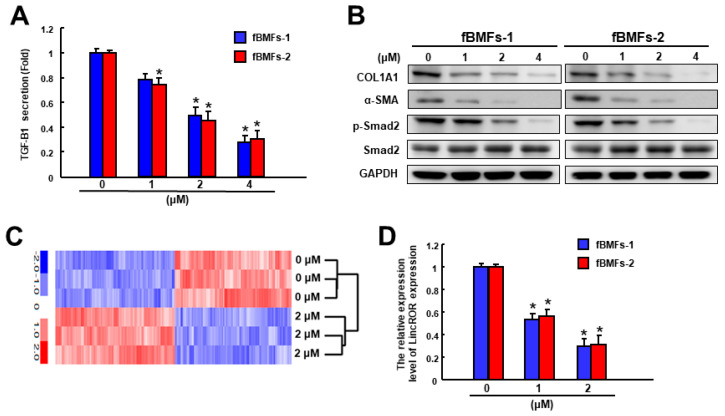
α-mangostin suppresses the expression of fibrosis markers, components of TGF-β signaling, and LincROR in fBMFs. (**A**) α-mangostin dose-dependently downregulated the secretion of TGF-β1 in fBMFs. (**B**) The protein expression levels of fibrosis (type I collagen) and myofibroblast markers (α-SMA) were dose-dependently downregulated along with the reduced phosphorylation of Smad2 by western blot. (**C**) The lncRNAs expression level of LincROR in the α-mangostin-treated fBMFs were analyzed by a high-throughput RNA sequencing approach. (**D**) qPCR analysis was applied to analyze the relative LincROR expression level in α-mangostin treated fBMFs. * *p* < 0.05 compared to the control.

A plethora of factors have been identified to regulate myofibroblasts, and TGF-β1 is the most notable stimulator of fibrosis. We showed that α-mangostin attenuated the production of TGF-β1 in fBMFs in a dose-dependent fashion (Figure 3A). α-SMA is a well-known myofibroblast marker and its expression upregulates contractile activity [37]. We observed the expression of α-SMA gradually decreased when α-mangostin was applied, suggesting the reduced numbers of myofibroblasts (Figure 3B). Type I collagen is the primary ECM protein deposited by myofibroblasts and it has been revealed that cells from OSF samples generated about 85% type I collagen and 15% type III collagen. Additionally, the ratio of its major components α1 (I) to α2 (I) chains was higher (3:1) in OSF cells than in normal fibroblasts (2:1) [38]. We found that α1 type I collagen (COL1A1) was suppressed by various concentrations of α-mangostin (Figure 3B). In accordance with the reduction of TGF-β1, the protein expression of phosphorylated Smad2 was downregulated as well (Figure 3B). Moreover, the result of RNA sequencing showed that the expression of LincROR was decreased (Figure 3C) and qRT-PCT analysis verified α-mangostin dose-dependently inhibited LincROR (Figure 3D).

### 3.4. The Inhibitory Property of α-Mangostin on Myofibroblast Activities and TGF-β Signaling Is Mediated by LincROR

**Figure 4:**
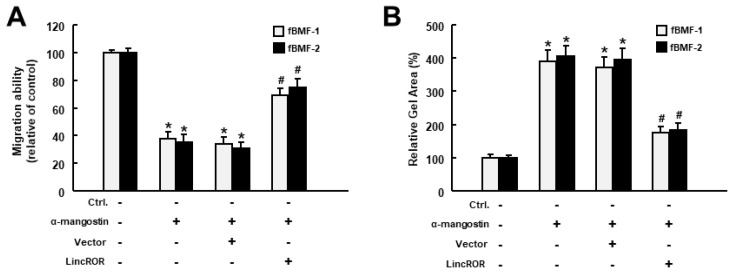

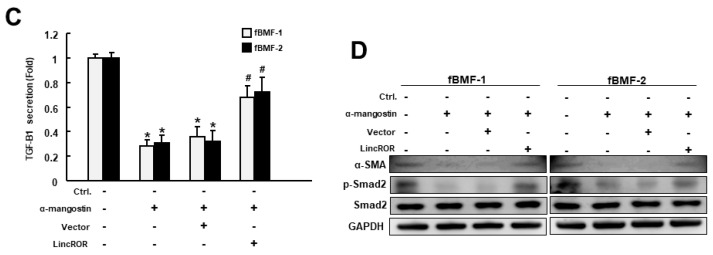
Ectopic expression of LincROR reverses the effects of α-mangostin on myofibroblast activities and TGF-β signaling. Administration of α-mangostin suppressed the transwell migration (**A**), collagen gel contraction (**B**), secretion of TGF-β1 (**C**) and protein expression of α-SMA, phosphorylated Smad2, and total Smad2 (**D**), whereas forced expression of LincROR abolished these effects. * *p* < 0.05 compared to control. ^#^ *p* < 0.05 compared to α-mangostin-only group.

To investigate whether LincROR was implicated in the suppressive effect of α-mangostin on myofibroblast activation, a transwell migration assay was used to show that forced expression of LincROR enhanced the migration capacity of the α-mangostin-treated fBMFs (Figure 4A). Likewise, ectopic expression of LincROR intensified the collagen gel contractility (Figure 4B) and TGF-β1 secretion (Figure 4C) compared to the fBMFs incubated with α-mangostin only. Moreover, the expression levels of α-SMA and phosphorylated Smad2 were re-increased in α-mangostin-treated fBMFs with overexpression of LincROR (Figure 4D). These results demonstrated that the elevation of LincROR counteracted the effects of α-mangostin on myofibroblast activities and TGF-β/Smad2 signaling.

### 3.5. LincROR Is Aberrantly Overexpressed in OSF Specimens 

**Figure 5:**
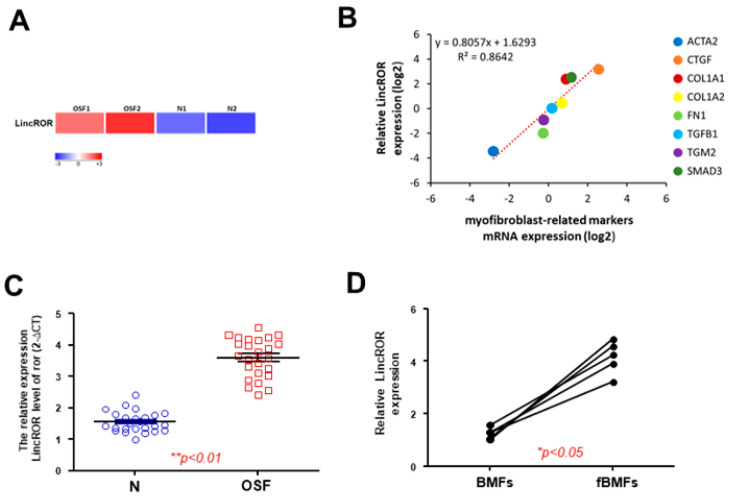
LincROR is upregulated in OSF specimens and positively correlated with various fibrosis markers. (**A**) A heatmap showing LincROR was highly expressed in two OSF tissues. (**B**) LincROR was positively associated with the indicated fibrosis-related markers. (**C**) Gene expression of LincROR in OSF samples and normal buccal mucosal tissues (*n* = 25). (**D**) Gene expression of LincROR in BMFs and fibrotic BMFs derived from OSF tissues. * *p* < 0.05 compared to control. ** *p* < 0.01.

After validating LincROR involved in the α-mangostin-mediated suppression of myofibroblast activities, we then assessed the expression of LincROR in OSF samples. As expected, LincROR was differentially expressed between OSF and normal tissues using RNA-Sequencing analysis (Figure 5A). In addition, the expression of LincROR was positively associated with numerous fibrosis-related markers, such as α-SMA (ACTA2), COL1A1, or TGF-β1 (TGFB1) (Figure 5B). To authenticate the result from RNA-Sequencing, qRT-PCR was conducted and showed that the expression of LincROR was elevated in OSF specimens (Figure 5C). Similarly, the expression of LincROR in fBMFs derived from OSF tissues was upregulated compared to normal BMFs (Figure 5D). 

### 3.6. Silencign of LincROR Inhibits Myofibroblast Activation

**Figure 6:**
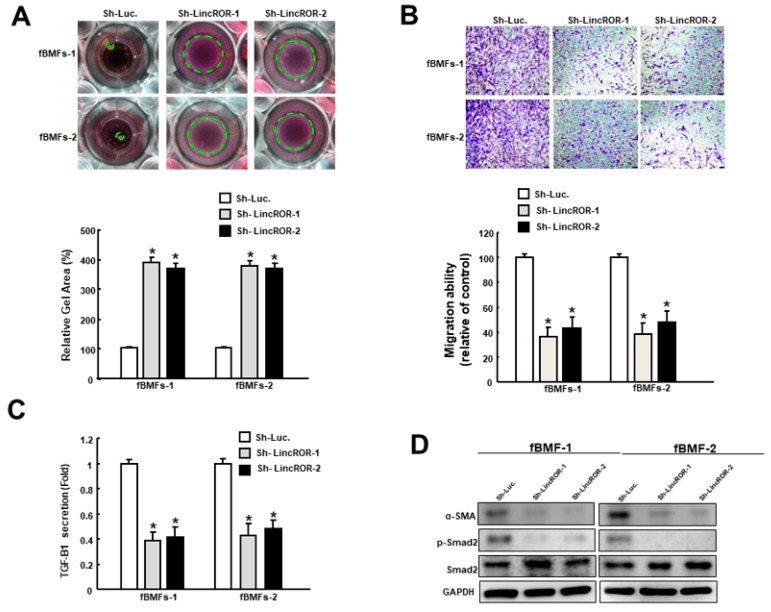
Silencing of LincROR downregulates myofibroblast features and fibrosis markers. Inhibition of LincROR diminished the collagen gel contraction (**A**), transwell migration (**B**), secretion of TGF-β1 (**C**), and protein expression of α-SMA, phosphorylated Smad2 and total Smad2 (**D**). * *p* < 0.05 compared to the control.

Subsequently, we investigated the functional role of LincROR in myofibroblast activation and found that suppression of LincROR relieved the collagen gel contraction ability in fBMFs (Figure 6A). Additionally, fBMFs with sh-LincROR displayed a significant reduction of cell migration capability (Figure 6B) and TGF-β1 production (Figure 6C). Moreover, the expression of α-SMA and phosphorylated Smad2 in fBMFs was decreased when LincROR was silenced (Figure 6D). In brief, these findings suggested the aberrantly overexpressed LincROR may contribute to the persistent activation of myofibroblasts in OSF. In addition, our results suggest that α-mangostin has an inhibitory effect on fBMFs via the regulation of LincROR.

## 4. Discussion

**Figure 7:**
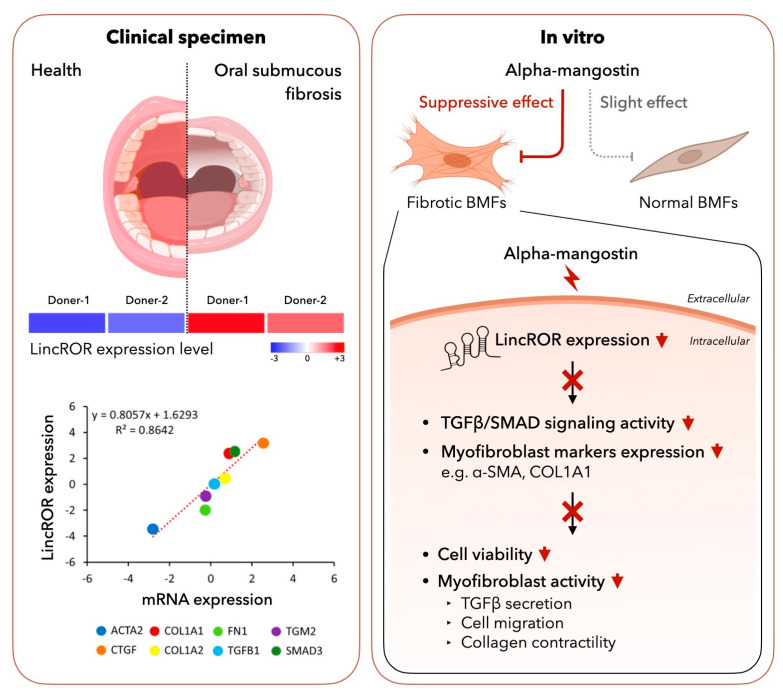
Schematic representation of the therapeutic potential of α-mangostin in OSF. LincROR is upregulated in OSF tissues using RNA-Seq and positively correlated with various fibrosis markers (**left** panel). α-mangostin inhibits myofibroblast characteristics, including cell proliferation, activities and marker expression as well as TGF-β1 signaling (**right** panel).

Several studies have demonstrated the pharmacological effects of α-mangostin, including antioxidant, anti-carcinogenic and anti-fibrosis activities. Here, we showed that α-mangostin exerted suppressive properties against OSF through the downregulation of lincROR. Our data revealed that the expression of lincROR was aberrantly upregulated in OSF specimens and positively correlated with various fibrosis factors, such as ACTA2, COL1A1 and fibronectin (FN1) (Figure 7). Administration of α-mangostin markedly attenuated the myofibroblast activation of fBMFs as evidenced by lower migratory and contractile capacities along with reduced expression of α-SMA and type I collagen. Suppression of TGF-β1/Smad2 signaling was in favor of our finding regarding the downregulation of myofibroblast activities, and these benefits may be due to the inhibition of lincROR by α-mangostin (Figure 7). Our results were in line with various studies showing that α-mangostin inhibited myofibroblast transdifferentiation and TGF-β-induced fibrotic response via suppressing nicotinamide adenine dinucleotide phosphate oxidase4 (NOX4)-generating reactive oxygen species (ROS) or enhancing antioxidant enzymes, leading to the alleviation of the liver [32], lung [30] or cardiac fibrosis [33]. Likewise, α-mangostin was demonstrated to reduce the expression of IL-6 and IL-8 expression in *P. gingivalis* LPS-stimulated human gingival fibroblasts [39]. It goes without saying that the anti-inflammatory and antioxidant features of α-mangostin contribute to its inhibitory ability of OSF since part of the pathogenesis of OSF is attributed to the elevation of inflammation and oxidative stress [40]. Our work further demonstrated that α-mangostin can mitigate fibrosis through the modulation of non-coding RNAs. Currently, only a limited number of studies have shown the relationship between α-mangostin and non-coding RNAs. For instance, α-mangostin has been found to restore the hyperglycemia-induced growth inhibition of human umbilical vein endothelial cells via regulation of lncRNA H19 [41]. Our results showed that administration of α-mangostin can modulate lincROR using fBMFs (myofibroblasts).

LincRoR is a 2.6 kb lncRNA located in chromosome 18 and was first identified in 2010 for its function as a key “Regulator of Reprogramming” [42]. Later, this pluripotency-associated lincRNA was often regarded as a carcinogenic factor as it was predominantly upregulated in various types of tumors, including oral cancer [34]. It has been shown that the expression of lincRoR was associated with several stemness-related genes, such as Oct4, Sox2, and Nanog. The promoter of the lincRoR gene contained the binding sites for Oct4, Sox2, and Nanog, and the transcription of lincRoR was activated upon binding of these transcriptional factors [42,43]. Apart from being a direct target of key pluripotency transcription factors, lincRoR also prevented these transcriptional factors from microRNA-145-mediated degradation. Accordingly, it has been suggested that lincRoR and these transcriptional factors may form an autoregulatory feedback loop during the self-renewal of embryonic stem cells [42,43]. In agreement with this finding, we showed that lincROR was abnormally overexpressed in precancerous OSF and fBMFs derived from OSF tissues. It also has been demonstrated that the expression levels of Oct4, Sox2, and Nanog were markedly elevated in tumor-adjacent tissues and may be associated with tumor progression of oral cancer [43]. Furthermore, a previous study has shown that chronic exposure of oral epithelial cells to arecoline led to upregulation of Oct4, Sox2, and Nanog as well as an increase in several EMT markers (e.g., Snail, Slug, and Twist) [44]. As such, the elevation of the abovementioned transcriptional factors following stimulation of arecoline may lead to the upregulation of lincRoR in OSF specimens and verification of this hypothesis is worthy of investigation in the future.

Considerable attention has been paid toward the emerging roles of lincRNAs in fibrogenesis, especially myofibroblast activation. LincRNAs may exert their modulatory property through the direct binding of target molecules or interaction with microRNAs. For instance, LINC00084 has been proven to function as a sponge of microRNA-204 and titrating the inhibition of microRNA-204 on EMT inducer zinc–finger E–box–binding 1 (ZEB1) in fBMFs [45]. Another study demonstrated that LINC00312 mediated myofibroblast activities via direct interaction of YBX1, a negative regulator of collagen expression [46]. Several studies also suggested that lincROR induces tumorigenesis via the regulation of EMT-associated factors. For example, lincRoR has been found to act as a competing endogenous RNA of microRNA-205, which prevented the degradation of EMT inducer ZEB2 and enhanced the aggressiveness of breast cancer cells [47]. Additionally, lincRoR promoted cell proliferation of pancreatic cancer through the elevation of ZEB1 [48]. Both ZEB1 and ZEB2 have been demonstrated to be implicated in myofibroblast transdifferentiation during OSF development [49,50]. Moreover, another EMT inducer twist family BHLH transcription factor (Twist) was found to be increased after arecoline treatment [51]. One of the recent studies has indicated that lincROR contributed to the chemoresistance of hepatocellular carcinoma through Twist-mediated EMT [52]. As a consequence, it is reasonable to postulate that the overexpressed lincROR may confer to the persistent activation of myofibroblasts through the mediation of EMT-associated factors, such as ZEB1, ZEB2 or Twist.

On the other hand, a number of microRNAs also hold the potential of acting as downstream mediators in the lincROR-associated fibrogenesis due to their predicted binding sites shared by lincROR, such as microRNA-145, microRNA-181 [43] and microRNA-205 [47]. LincRoR has been shown to modulate cancer progression via interaction with microRNA-145 [47], and microRNA-145-5p was shown to ameliorate hypertrophic scar through suppression of myofibroblast activation and reduction of Smad2/3 [53]. Furthermore, microRNA-181a has been demonstrated as an anti-fibrotic factor in fBMFs [54], and participated in TGF-β-induced EMT in hepatocytes [55]. As for miR-205, it was found to attenuate the angiotensin II-induced fibrosis in vivo and myofibroblast activation in atrial fibroblasts [56]. Given that we observed the decreased myofibroblast activities and TGF-β/Smad2 signaling in fBMFs with sh-lincROR, whether lincROR contributes to the development of OSF via functioning as a competing endogenous RNA of microRNA-145, microRNA-181, or miR-205 requires further investigation.

Altogether, our data showed that administration of α-mangostin may alleviate the persistent activation of myofibroblasts through inhibition of the TGF-β/Smad2 pathway and downregulation of the aberrantly overexpressed lincROR. These results suggested that α-mangostin-containing foods may be good nutritional supplements for OSF patients.

## Data Availability

Not applicable.

## References

[1] Iocca O., Sollecito T.P., Alawi F., Weinstein G.S., Newman J.G., De Virgilio A., Di Maio P., Spriano G., Pardiñas López S., Shanti R.M. (2020). Potentially malignant disorders of the oral cavity and oral dysplasia: A systematic review and meta-analysis of malignant transformation rate by subtype. Head Neck.

[2] Wang Y.-Y., Tail Y.-H., Wang W.-C., Chen C.-Y., Kao Y.-H., Chen Y.-K., Chen C.-H. (2014). Malignant transformation in 5071 southern Taiwanese patients with potentially malignant oral mucosal disorders. BMC Oral Health.

[3] Chiu C.-J., Chang M.-L., Chiang C.-P., Hahn L.-J., Hsieh L.-L., Chen C.-J. (2002). Interaction of collagen-related genes and susceptibility to betel quid-induced oral submucous fibrosis. Cancer Epidemiol. Biomark. Prev..

[4] Elamin F., Steingrimsdottir H., Wanakulasuriya S., Johnson N., Tavassoli M. (1998). Prevalence of human papillomavirus infection in premalignant and malignant lesions of the oral cavity in U.K. subjects: A novel method of detection. Oral Oncol..

[5] Lee C.-H., Ko Y.-C., Huang H.-L., Chao Y.-Y., Tsai C.-C., Shieh T.-Y., Lin L.-M. (2003). The precancer risk of betel quid chewing, tobacco use and alcohol consumption in oral leukoplakia and oral submucous fibrosis in southern Taiwan. Br. J. Cancer.

[6] Tsai C.C., Ma R.H., Shieh T.Y. (1999). Deficiency in collagen and fibronectin phagocytosis by human buccal mucosa fibroblasts in vitro as a possible mechanism for oral submucous fibrosis. J. Oral Pathol. Med..

[7] Shieh D.-H., Chiang L.-C., Shieh T.-Y. (2003). Augmented mRNA expression of tissue inhibitor of metalloproteinase-1 in buccal mucosal fibroblasts by arecoline and safrole as a possible pathogenesis for oral submucous fibrosis. Oral Oncol..

[8] Chang Y.-C., Yang S.-F., Tai K.-W., Chou M.-Y., Hsieh Y.-S. (2002). Increased tissue inhibitor of metalloproteinase-1 expression and inhibition of gelatinase A activity in buccal mucosal fibroblasts by arecoline as possible mechanisms for oral submucous fibrosis. Oral Oncol..

[9] Khan I., Kumar N., Pant I., Narra S., Kondaiah P. (2012). Activation of TGF-β Pathway by Areca Nut Constituents: A Possible Cause of Oral Submucous Fibrosis. PLoS ONE.

[10] Pant I., Kumar N., Khan I., Rao S.G., Kondaiah P. (2015). Role of Areca Nut Induced TGF-β and Epithelial-Mesenchymal Interaction in the Pathogenesis of Oral Submucous Fibrosis. PLoS ONE.

[11] Hinz B., Phan S.H., Thannickal V.J., Galli A., Bochaton-Piallat M.-L., Gabbiani G. (2007). The Myofibroblast: One Function, Multiple Origins. Am. J. Pathol..

[12] Iwaisako K., Jiang C., Zhang M., Cong M., Moore-Morris T.J., Park T.J., Liu X., Xu J., Wang P., Paik Y.-H. (2014). Origin of myofibroblasts in the fibrotic liver in mice. Proc. Natl. Acad. Sci. USA.

[13] LeBleu V.S., Taduri G., O’Connell J., Teng Y., Cooke V.G., Woda C., Sugimoto H., Kalluri R. (2013). Origin and function of myofibroblasts in kidney fibrosis. Nat. Med..

[14] Thiery J.P., Acloque H., Huang R.Y., Nieto M.A. (2009). Epithelial-Mesenchymal Transitions in Development and Disease. Cell.

[15] Yang H.-W., Lu M.-Y., Chiu Y.-W., Liao Y.-W., Huang Y.-F., Ju Chueh P., Hsieh P.-L., Yu C.-C. (2018). Hinokitiol ablates myofibroblast activation in precancerous oral submucous fibrosis by targeting Snail. Environ. Toxicol..

[16] Fang C.Y., Hsia S.M., Hsieh P.L., Liao Y.W., Peng C.Y., Wu C.Z., Lin K.C., Tsai L.L., Yu C. (2019). Slug mediates myofibroblastic differentiation to promote fibrogenesis in buccal mucosa. J. Cell. Physiol..

[17] Peng C.-Y., Liao Y.-W., Lu M.-Y., Yang C.-M., Hsieh P.-L., Yu C.-C. (2020). Positive Feedback Loop of SNAIL-IL-6 Mediates Myofibroblastic Differentiation Activity in Precancerous Oral Submucous Fibrosis. Cancers.

[18] Lee P.-H., Chu P.-M., Hsieh P.-L., Yang H.-W., Chueh P.J., Huang Y.-F., Liao Y.-W., Yu C.-C. (2018). Glabridin inhibits the activation of myofibroblasts in human fibrotic buccal mucosal fibroblasts through TGF-β/smad signaling. Environ. Toxicol..

[19] Chen P.-Y., Ho D.C., Liao Y.-W., Hsieh P.-L., Lu K.-H., Tsai L.-L., Su S.-H., Yu C.-C. (2021). Honokiol inhibits arecoline-induced oral fibrogenesis through transforming growth factor-β/Smad2/3 signaling inhibition. J. Formos. Med. Assoc..

[20] Gu S., Jin L., Zhang F., Sarnow P., Kay M.A. (2009). Biological basis for restriction of microRNA targets to the 3′ untranslated region in mammalian mRNAs. Nat. Struct. Mol. Biol..

[21] Kopp F., Mendell J.T. (2018). Functional Classification and Experimental Dissection of Long Noncoding RNAs. Cell.

[22] Salmena L., Poliseno L., Tay Y., Kats L., Pandolfi P.P. (2011). A ceRNA Hypothesis: The Rosetta Stone of a Hidden RNA Language?. Cell.

[23] Hsieh P.-L., Chen S.-H., Huang Y.-F., Lu M.-Y., Yu C.-C. (2022). The functional roles of microRNAs in the pathogenesis of oral submucous fibrosis. J. Dent. Sci..

[24] Yu C.-C., Liao Y.-W., Hsieh P.-L., Chang Y.-C. (2021). Targeting lncRNA H19/miR-29b/COL1A1 Axis Impedes Myofibroblast Activities of Precancerous Oral Submucous Fibrosis. Int. J. Mol. Sci..

[25] Fang C.Y., Yu C.C., Liao Y.W., Hsieh P.L., Lu M.Y., Lin K.C., Wu C.Z., Tsai L.L. (2019). LncRNA LINC00974 activates TGF-β/Smad signaling to promote oral fibrogenesis. J. Oral Pathol. Med..

[26] Lin C.-Y., Hsieh P.-L., Liao Y.-W., Peng C.-Y., Yu C.-C., Lu M.-Y. (2019). Arctigenin Reduces Myofibroblast Activities in Oral Submucous Fibrosis by LINC00974 Inhibition. Int. J. Mol. Sci..

[27] Ransohoff J.D., Wei Y., Khavari P.A. (2018). The functions and unique features of long intergenic non-coding RNA. Nat. Rev. Mol. Cell Biol..

[28] Kwak H.-H., Kim I.-R., Kim H.-J., Park B.-S., Yu S.-B. (2016). *α*-Mangostin Induces Apoptosis and Cell Cycle Arrest in Oral Squamous Cell Carcinoma Cell. Evid.-Based Complement. Altern. Med..

[29] Tangsuksan P., Chuerduangphui J., Takahashi Yupanqui C., Srichana T., Hitakomate E., Pientong C., Ekalaksananan T., Nittayananta W. (2021). Mucoadhesive film containing α-mangostin shows potential role in oral cancer treatment. BMC Oral Health.

[30] Li R.S., Xu G.-H., Cao J., Liu B., Xie H.-F., Ishii Y., Zhang C.-F. (2019). Alpha-Mangostin Ameliorates Bleomycin-Induced Pulmonary Fibrosis in Mice Partly through Activating Adenosine 5′-Monophosphate-Activated Protein Kinase. Front. Pharmacol..

[31] Soetikno V., Murwantara A., Andini P., Charlie F., Lazarus G., Louisa M., Arozal W. (2020). Alpha-Mangostin Improves Cardiac Hypertrophy and Fibrosis and Associated Biochemical Parameters in High-Fat/High-Glucose Diet and Low-Dose Streptozotocin Injection-Induced Type 2 Diabetic Rats. J. Exp. Pharmacol..

[32] Lestari N., Louisa M., Soetikno V., Suwana A.G., Ramadhan P.A., Akmal T., Arozal W. (2018). Alpha Mangostin Inhibits the Proliferation and Activation of Acetaldehyde Induced Hepatic Stellate Cells through TGF-β and ERK 1/2 Pathways. J. Toxicol..

[33] Sari N., Katanasaka Y., Sugiyama Y., Miyazaki Y., Sunagawa Y., Funamoto M., Shimizu K., Shimizu S., Hasegawa K., Morimoto T. (2021). Alpha Mangostin Derived from Garcinia magostana Linn Ameliorates Cardiomyocyte Hypertrophy and Fibroblast Phenotypes in Vitro. Biol. Pharm. Bull..

[34] Arunkumar G., Deva Magendhra Rao A.K., Manikandan M., Arun K., Vinothkumar V., Revathidevi S., Rajkumar K.S., Rajaraman R., Munirajan A.K. (2017). Expression profiling of long non-coding RNA identifies linc-RoR as a prognostic biomarker in oral cancer. Tumor Biol..

[35] Liao Y.-W., Tsai L.-L., Lee Y.-H., Hsieh P.-L., Yu C.-C., Lu M.-Y. (2022). miR-21 promotes the fibrotic properties in oral mucosa through targeting PDCD4. J. Dent. Sci..

[36] Bell E., Ivarsson B., Merrill C. (1979). Production of a tissue-like structure by contraction of collagen lattices by human fibroblasts of different proliferative potential in vitro. Proc. Natl. Acad. Sci. USA.

[37] Hinz B., Celetta G., Tomasek J.J., Gabbiani G., Chaponnier C. (2001). Alpha-Smooth Muscle Actin Expression Upregulates Fibroblast Contractile Activity. Mol. Biol. Cell.

[38] Kuo M.Y., Chen H.M., Hahn L.J., Hsieh C.C., Chiang C.-P. (1995). Collagen Biosynthesis in Human Oral Submucous Fibrosis Fibroblast Cultures. J. Dent. Res..

[39] Yiemwattana I., Kaomongkolgit R. (2015). Alpha-mangostin suppresses IL-6 and IL-8 expression in P. gingivalis LPS-stimulated human gingival fibroblasts. Odontology.

[40] Shih Y.-H., Wang T.-H., Shieh T.-M., Tseng Y.-H. (2019). Oral Submucous Fibrosis: A Review on Etiopathogenesis, Diagnosis, and Therapy. Int. J. Mol. Sci..

[41] Luo Y., Fang Z., Ling Y., Luo W. (2019). LncRNA-H19 acts as a ceRNA to regulate HE4 expression by sponging miR-140 in human umbilical vein endothelial cells under hyperglycemia with or without α-Mangostin. Biomed. Pharmacother..

[42] Loewer S., Cabili M.N., Guttman M., Loh Y.-H., Thomas K., Park I.H., Garber M., Curran M., Onder T., Agarwal S. (2010). Large intergenic non-coding RNA-RoR modulates reprogramming of human induced pluripotent stem cells. Nat. Genet..

[43] Wang Y., Xu Z., Jiang J., Xu C., Kang J., Xiao L., Wu M., Xiong J., Guo X., Liu H. (2013). Endogenous miRNA Sponge lincRNA-RoR Regulates Oct4, Nanog, and Sox2 in Human Embryonic Stem Cell Self-Renewal. Dev. Cell.

[44] Wang T.Y., Peng C.-Y., Lee S.-S., Chou M.-Y., Yu C.-C., Chang Y.-C. (2016). Acquisition cancer stemness, mesenchymal transdifferentiation, and chemoresistance properties by chronic exposure of oral epithelial cells to arecoline. Oncotarget.

[45] Lee Y.-H., Liao Y.-W., Lu M.-Y., Hsieh P.-L., Yu C.-C. (2021). LINC00084/miR-204/ZEB1 Axis Mediates Myofibroblastic Differentiation Activity in Fibrotic Buccal Mucosa Fibroblasts: Therapeutic Target for Oral Submucous Fibrosis. J. Pers. Med..

[46] Yu C.-H., Fang C.-Y., Yu C.-C., Hsieh P.-L., Liao Y.-W., Tsai L.-L., Chu P.-M. (2020). LINC00312/YBX1 Axis Regulates Myofibroblast Activities in Oral Submucous Fibrosis. Int. J. Mol. Sci..

[47] Hou P., Zhao Y., Li Z., Yao R., Ma M., Gao Y., Zhao L., Zhang Y., Huang B., Lu J. (2014). LincRNA-ROR induces epithelial-to-mesenchymal transition and contributes to breast cancer tumorigenesis and metastasis. Cell Death Dis..

[48] Zhan H.-X., Wang Y., Li C., Xu J.-W., Zhou B., Zhu J.-K., Han H.-F., Wang L., Wang Y.-S., Hu S.-Y. (2016). LincRNA-ROR promotes invasion, metastasis and tumor growth in pancreatic cancer through activating ZEB1 pathway. Cancer Lett..

[49] Chang Y.C., Tsai C.H., Lai Y.L., Yu C.C., Chi W.Y., Li J.J., Chang W.W. (2014). Arecoline-induced myofibroblast transdifferentiation from human buccal mucosal fibroblasts is mediated by ZEB 1. J. Cell. Mol. Med..

[50] Liao Y.-W., Yu C.-C., Hsieh P.-L., Chang Y.-C. (2018). miR-200b ameliorates myofibroblast transdifferentiation in precancerous oral submucous fibrosis through targeting ZEB 2. J. Cell. Mol. Med..

[51] Lee Y.-H., Yang L.-C., Hu F.-W., Peng C.-Y., Yu C.-H., Yu C.-C. (2016). Elevation of Twist expression by arecoline contributes to the pathogenesis of oral submucous fibrosis. J. Formos. Med. Assoc..

[52] Zhang Y., Wu W., Sun Q., Ye L., Zhou D., Wang W. (2020). linc-ROR facilitates hepatocellular carcinoma resistance to doxorubicin by regulating TWIST1-mediated epithelial-mesenchymal transition. Mol. Med. Rep..

[53] Shen W., Wang Y., Wang D., Zhou H., Zhang H., Li L. (2019). miR-145-5p attenuates hypertrophic scar via reducing Smad2/Smad3 expression. Biochem. Biophys. Res. Commun..

[54] Fang C.-Y., Chen S.-H., Huang C.-C., Liao Y.-W., Chao S.-C., Yu C.-C. (2022). Fucoidan-Mediated Inhibition of Fibrotic Properties in Oral Submucous Fibrosis via the MEG3/miR-181a/Egr1 Axis. Pharmaceuticals.

[55] Brockhausen J., Tay S.S., Grzelak C.A., Bertolino P., Bowen D.G., D’Avigdor W.M., Teoh N., Pok S., Shackel N., Gamble J.R. (2015). miR-181a mediates TGF-β-induced hepatocyte EMT and is dysregulated in cirrhosis and hepatocellular cancer. Liver Int..

[56] Xiao Z., Reddy D.P.K., Xue C., Liu X., Chen X., Li J., Ling X., Zheng S. (2021). Profiling of miR-205/P4HA3 following Angiotensin II-Induced Atrial Fibrosis: Implications for Atrial Fibrillation. Front. Cardiovasc. Med..

